# Nicotinamide Mononucleotide: Research Process in Cardiovascular Diseases

**DOI:** 10.3390/ijms25179526

**Published:** 2024-09-02

**Authors:** Haoyuan Deng, Ding Ding, Yu Ma, Hao Zhang, Ningning Wang, Cong Zhang, Guang Yang

**Affiliations:** 1 Department of Nutrition and Food Hygiene, School of Public Health, Dalian Medical University, Dalian 116044, China; zkxwnn@dmu.edu.cn (N.W.); congzhang1203@hotmail.com (C.Z.); yg290@dmu.edu.cn (G.Y.); 2 School of Public Health, Dalian Medical University, Dalian 116044, China; dingdingmail0000@126.com (D.D.); ahao112@outlook.com (H.Z.); 3 Department of Health Toxicology, School of Public Health, Dalian Medical University, Dalian 116044, China; may11@dmu.edu.cn

**Keywords:** nicotinamide mononucleotide, nicotinamide mononucleotide supplementation, cardiovascular diseases, NAD^+^

## Abstract

Nicotinamide adenine dinucleotide (NAD^+^) is an essential metabolite that plays a crucial role in diverse biological processes, including energy metabolism, gene expression, DNA repair, and mitochondrial function. An aberrant NAD^+^ level mediates the development of cardiovascular dysfunction and diseases. Both in vivo and in vitro studies have demonstrated that nicotinamide mononucleotide (NMN), as a NAD^+^ precursor, alleviates the development of cardiovascular diseases such as heart failure, atherosclerosis, and myocardial ischemia/reperfusion injury. Importantly, NMN has suggested pharmacological activities mostly through its involvement in NAD^+^ biosynthesis. Several clinical studies have been conducted to investigate the efficacy and safety of NMN supplementation, indicating its potential role in cardiovascular protection without significant adverse effects. In this review, we systematically summarize the impact of NMN as a nutraceutical and potential therapeutic drug on cardiovascular diseases and emphasize the correlation between NMN supplementation and cardiovascular protection.

## 1. Introduction

Nicotinamide adenine dinucleotide (NAD^+^) serves as an essential cosubstrate in the regulation of metabolism, gene expression, DNA repair, mitochondrial function, and other diverse biological processes [1]. NAD^+^ has been identified as a critical metabolic redox co-enzyme involved in enzymatic reactions [2] that plays a crucial role in health-span and life-span. The loss of NAD^+^ promotes the occurrence of cardiovascular diseases, especially cardiovascular dysfunction and remodeling, such as atrial fibrillation (AF), heart failure (HF), and ischemia/reperfusion (I/R) injury [3,4,5]. Thus, NAD^+^ is an effective therapeutic target against cardiovascular diseases.

Nicotinamide mononucleotide (NMN), an intermediate product in NAD^+^ biosynthesis, is formed by the reaction between a phosphate group and a nucleoside containing ribose and nicotinamide [6]. Nicotinamide (NAM) is converted to NMN by nicotinamide phosphoribosyltransferase (NAMPT). NMN is widely abundant in various natural foods, including broccoli, cabbage, avocado, tomato, raw beef, and milk [7,8]. NMN also has been considered as a source of energy related to metabolism and energy production, including ATP synthesis. In the biosynthetic process of NAD^+^, NMN, as a key substrate for enzymes such as nicotinamide mononucleotide adenylyltransferase 1 (NMNAT 1) of nuclear origin or NMNAT 3 of mitochondrial origin, contributes to the enzymatic conversion to NAD^+^ in humans [9]. Moreover, it has been demonstrated that NMN supplementation represents multiple pharmacological effects on cardiovascular and cerebrovascular diseases, Alzheimer’s disease, obesity, and diabetes, which are closely related to the loss of NAD^+^ [10,11,12].

This mini-review mainly describes the role of NMN in NAD^+^ biosynthesis and will focus on its cardiovascular protective efficacy. As a nutraceutical, the safety and efficiency of the oral administration of NMN in humans will also be discussed.

## 2. The Pathway of NMN Biosynthesis and Absorption

NMN is an intermediate product in the process of NAD^+^ biosynthesis. Thus, to better understand the function of NMN, we firstly focus on the pathway of NAD^+^ biosynthesis. Previous studies have suggested that intracellular NAD^+^ is mostly generated by the salvage pathway. In this pathway, NAM is converted into NMN by NAMPT catalysis. Then, NMN is finally converted to NAD^+^ via NMNAT [13,14,15]. Meanwhile, NAD^+^ is degraded to NAM by NAD^+^-consuming enzymes (Sirts, PARPs, CD38, and CD157), followed by its conversion to NMN through the catalysis of NAMPT, and this process is essential for maintaining NAD^+^ homeostasis [16].

**Figure 1:**
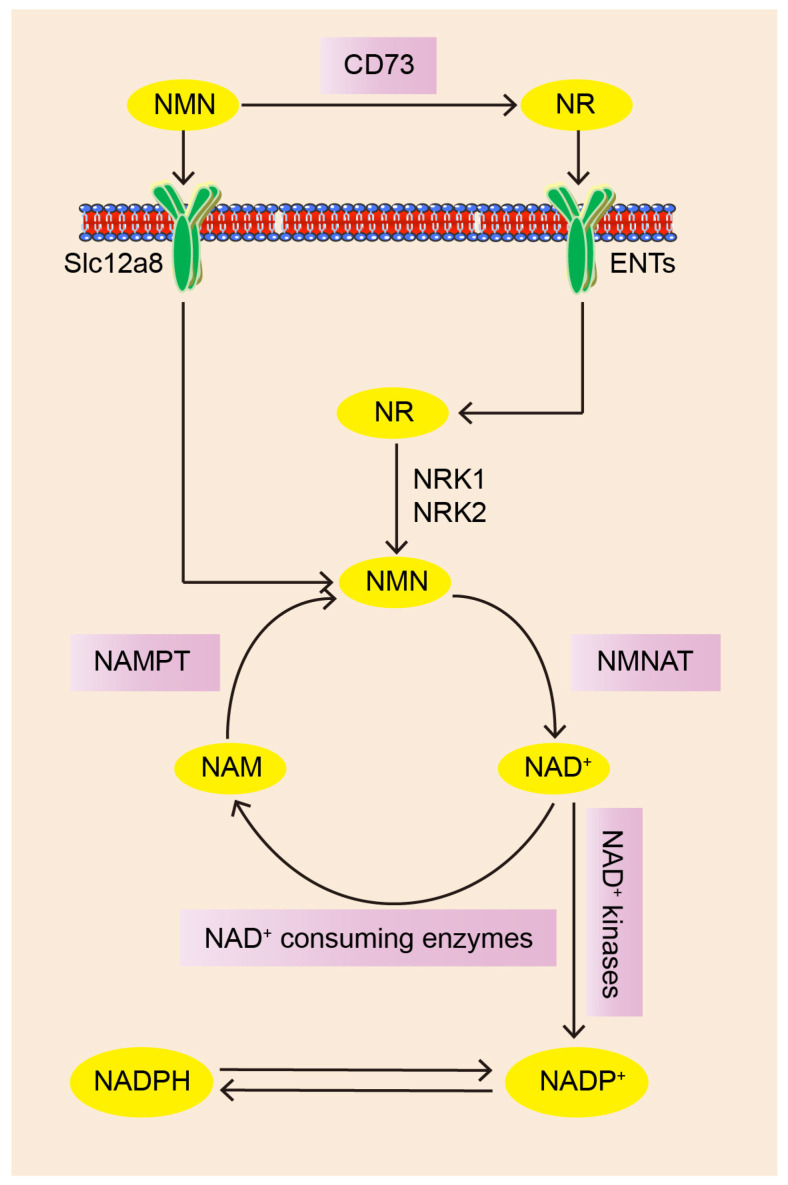
The pathway of NMN to NAD^+^ biosynthesis. The salvage pathway is mainly the manner for intracellular NAD^+^ biosynthesis. The degradation of NAD^+^ to NAM replenishes the intracellular NAD^+^ levels, which is essential for maintaining NAD^+^ homeostasis. Exogenous NMN enters into cells depending on two ways, including the NR-NRK pathway and Slc12a8 transporter, to participate in NAD^+^ biosynthesis. NMN: nicotinamide mononucleotide; NAD^+^: nicotinamide adenine dinucleotide; NAM: nicotinamide; NR: nicotinamide riboside; NMNAT: nicotinamide mononucleotide adenylyl transferase; NAMPT: nicotinamide phosphoribosyl transferase; NRK1: nicotinamide riboside kinase 1; NRK2: nicotinamide riboside kinase 2; ENTs: equilibrative nucleoside transporters; NADP^+^: nicotinamide adenine dinucleotide phosphate.

Exogenous NMN for synthesizing NAD^+^ initially needs to be transported into mammalian cells. Nicotinamide riboside (NR) is another NAD^+^ precursor molecule. Exogenous NMN is firstly converted to NR with the help of CD73, and then it is absorbed by cells through equilibrative nucleoside transporters (ENTs). NR is phosphorylated to generate NMN by the activity of nicotinamide riboside kinases (NRKs), and then NMN is converted into NAD^+^ [6,17]. NRKs are highly conserved enzymes in all eukaryotes and are encoded by the *Nmrk* genes [6]. NRK1 and NRK2 are two types of enzymes of the NRK protein family in mammals. Despite NRK1 and NRK2 representing similar activity, the two enzymes exhibit tissue-specific expression. NRK1 is ubiquitously expressed in various tissues, whereas NRK2 is specifically expressed in skeletal muscle and the myocardium. NRK1 is a necessary and rate-limiting enzyme for the conversion of exogenous NMN to NAD^+^ [18]. NRK2 knockdown significantly diminishes the NAD^+^ level in the heart, thereby highlighting the pivotal role of NRK2 in maintaining cardiac NAD^+^ levels [19]. The *Slc12a8* gene is highly expressed in the pancreas and small intestine and moderately expressed in the white adipose tissue and liver, and it encodes a cell membrane-specific transporter that directly mediates NMN transportation through a sodium-dependent mechanism [20]. This manner promotes the oral administration of NMN absorption rapidly through the intestinal tract and leads to plasma NMN level elevation [7] (Figure 1).

NMN is rapidly absorbed from the gastrointestinal tract into the blood and finally enters into various tissues. Subsequently, NMN is converted into NAD^+^ and accumulated in these tissues. The intraperitoneal administration of NMN (500 mg/kg) increases the NMN and NAD^+^ levels in the liver, pancreas, and white adipose tissue (WAT) within 15 min in wild-type mice [12]. Similarly, the oral administration of NMN at a dosage of 300 mg/kg to mice significantly increases their plasma NMN levels at 2.5 min, indicating the rapid absorption of NMN in the gastrointestinal tract. Consistent with the changes, the concentration of NAD^+^ increases from 15 to 30 min in the liver [7].

Because of the crucial role of NMN in the process of NAD^+^ biosynthesis, elucidating the pathway of NMN synthesis and absorption facilitates its precise application.

## 3. NMN in Cardiovascular Diseases

Recently, the role of NMN in anti-ageing and life-span has been well demonstrated, and it has been considered as a potential therapeutic candidate for various diseases [21]. The major pharmacological function of NMN is the promotion of NAD^+^ biosynthesis, whereas a direct high dosage of NAD^+^ supplementation leads to diverse side effects, such as insomnia, fatigue, and anxiety. Moreover, the penetration capability of NAD^+^ across the plasma membrane is limited compared to NMN [22]. Importantly, NMN supplementation has numerous beneficial effects on various diseases, such as cardiovascular diseases [23].

### 3.1. Myocardial Ischemia/Reperfusion Injury

Ischemia is characterized by the reduction in the oxygen and ATP levels in tissues, leading to necrosis. Reperfusion refers to the process of reintroducing blood into previously ischemic tissues, which often results in calcium overload and reactive oxygen species (ROS) accumulation [24]. Ischemia followed by reperfusion results in the severe damage of tissues. Myocardial I/R injury refers to the tissue damage resulting from the restoration of the blood supply in myocardial tissue following a period of ischemia and leads to potential life-threatening clinical complications [25].

The activation of Sirt1 confers cardiovascular protection against I/R-induced injury [26]. Sirt1 promotes anti-oxidants and anti-apoptotic factor expression, including MnSOD, Trx1, and Bcl-xL, and decreases the expression of pro-apoptotic factors, such as Bax and cleaved Caspase-3, thereby augmenting cardiac resistance to oxidative stress and apoptosis [26]. The Sirt1 promotion of the MnSOD level is partially mediated by Foxo1, a transcription factor deacetylated by Sirt1. Meanwhile, the deacetylase activity of Sirt1 relies on NAD^+^. Ischemic preconditioning (IPC) is a potent endogenous mechanism that confers protection against myocardial I/R injury. Enhancing the NAD^+^ level facilitates Sirt1-mediated IPC. NAMPT plays a critical role in regulating the NAD^+^ and ATP levels, thereby emphasizing the significance of maintaining NAMPT expression to mitigate I/R-induced myocardial injury [27]. It has been reported that IPC promotes NAMPT expression, and the cardiovascular protective effect of IPC against I/R injury has been found to be attenuated in NAMPT^+/−^ mice, indicating the essential role of NAMPT in mediating this protective effect [10].

NMN exhibits a cardiovascular protective effect in the context of myocardial I/R injury. The intraperitoneal administration of NMN significantly elevates the baseline level of NAD^+^ in the heart and effectively prevents its decline during ischemia. Moreover, exogenous NMN exhibits a cardiovascular protective effect against I/R injury when administered once 30 min prior to ischemia or four times immediately before and during reperfusion, indicating its efficacy in safeguarding the heart throughout both the ischemic and reperfusion phases [10]. The cardiovascular protective effect of NMN is accompanied by a reduction in Foxo1 acetylation, whereas this effect is not prominently observed in Sirt1 knockout mice, indicating that this protective effect of NMN is mediated through the activation of Sirt1 [10]. NMN supplementation attenuates the I/R-induced myocardial infarct size in aged rats (22–24 months). Notably, NMN treatment increases the expression of Bcl-2, Sirt3, and Foxo1, while it decreases the expression of Bax and Caspase-3, compared to the I/R group [28]. These results suggest that NMN exerts a cardiovascular protective effect against myocardial I/R injury by mitigating apoptosis and activating the Sirt3/Foxo1 signaling pathway. Furthermore, NMN supplementation improves the myocardial hemodynamic parameters and reduces the release of lactate dehydrogenase in aged rats. NMN supplementation effectively mitigates cardiac oxidative stress and the mitochondrial ROS level, enhances the mitochondrial membrane potential, and restores the NAD^+^/NADH ratio [29]. These findings demonstrate that NMN improves mitochondrial function, reduces oxidative stress, and enhances the anti-oxidant defense system to protect against myocardial I/R injury.

Glycolysis is a metabolic pathway, and it mediates the development of oxidative stress and apoptosis [30]. Moreover, glycolysis has been implicated in the process of cardiovascular protection. A nutrient-sensitized screening for drugs enhancing glycolysis yielded hits that exhibit a cardiovascular protective effect in I/R injury models [31]. IPC modulates multiple metabolic pathways, including the promotion of glycolysis and glycogen synthesis [32]. I/R injury mediates the opening of the mitochondrial permeability transition (PT) pores. The closed state of PT pores is maintained during cardiac ischemia under acidic pH conditions, whereas change in the pH during reperfusion promotes the opening of mitochondrial PT pores [33]. Furthermore, the pH hypothesis of postconditioning posits that increasing acidic media confers cardiovascular protection by preserving the closed state of PT pores in early reperfusion [34]. Importantly, glycolysis has been proposed as a potential mechanism of NMN-mediated cardiovascular protection. NMN stimulates glycolysis and enhances ATP production during ischemia, and it elevates cardiac lactate and pyruvate to induce acidosis during reperfusion, thereby conferring protection against myocardial I/R-induced injury [35].

### 3.2. Heart Failure

HF is the late stage in the progression of various cardiovascular diseases, and it is a leading cause of morbidity and mortality worldwide. Mitochondrial dysfunction is implicated in the pathogenesis of HF. The deletion of Ndufs4, a critical protein for complex I (C-I) assembly, results in complex I-supported respiration decreases in the heart. Cardiac-specific Ndufs4 knockout (cKO) mice exhibit without significant alterations in cardiac function, energy, and longevity under unstressed conditions. However, Ndufs4 cKO mice with complex I deficiency demonstrate an accelerated heart failure following pressure overload or repeated pregnancy. The deficiency of complex I significantly reduces the NAD^+^/NADH ratio, thereby inhibiting Sirt3 activity and resulting in an increase in mitochondrial protein acetylation and sensitized permeability transition in mitochondria (mPTP). Additionally, NMN supplementation to Ndufs4 cKO mice partially restores the NAD^+^/NADH ratio, protein acetylation, and mPTP sensitivity [36]. Elevation of the NADH/NAD^+^ ratio stimulates mitochondrial protein hyperacetylation, leading to the development of HF [37]. Cardiac-specific KLF4 knockout mice promote mitochondrial protein hyperacetylation, rendering the mitochondria and heart more susceptible to stress. The Sirt3, NAD^+^, and NAMPT levels are decreased in the KLF4-deficient mouse heart. More important, the short-term administration of NMN effectively maintains mitochondrial homeostasis, reduces ROS production, prevents cardiac cell death, and ameliorates heart function in pressure overload-induced HF [5]. Remarkably, long-chain Acyl-CoA dehydrogenase (LCAD), a key fatty acid oxidation enzyme, catalyzes the oxidative metabolism of long-chain fatty acids, which serve as the primary energy source of the heart. It has been suggested that Sirt3 modulates mitochondrial fatty acid metabolism by mediating the enzymatic activity of LCAD, while NMN supplementation enhances mitochondrial fatty acid oxidation enzymes, indicating that NMN improves the cardiac energetic and heart function [5,38].

### 3.3. Atherosclerosis

Atherosclerosis is characterized by vascular dysfunction and abnormal lipid metabolism, and it takes several decades until the clinical complications occur in humans [39]. Inflammation is implicated in the pathogenesis of atherosclerosis. Macrophages mediate the development of inflammation by generating various cytokines and growth factors [40]. Activated M1 macrophages promote inflammation through the secretion of cytokines, including interleukin-1 beta (IL-1β), interleukin-6 (IL-6), tumor necrosis factor-alpha (TNF-α), and prostaglandins (PGE2) [41]. PGE2 mediates the inflammatory response, and it is synthesized by cyclooxygenases (COX-1 and COX-2), which serve as the targets of anti-inflammation [42]. Activated M1 macrophages significantly reduce the NAD^+^/NADPH ratio, whereas NMN supplementation increases the NAD^+^ level and decreases the production of cytokines (IL-1β and IL-6) in LPS-induced macrophages. NMN treatment decreases the LPS-induced responsive protein level associated with the pathway of acute-phase response signaling, LXR/RXR activation, FXR/RXR activation, and the complement system. Notably, NMN treatment significantly attenuates the LPS-induced expression of COX-2 in macrophages. Consequently, PGE2 expression is remarkably reduced with NMN supplementation in LPS-treated macrophages, indicating that NMN inactivates macrophages via the COX-2-PGE2 signaling pathway. These results have been validated in activated THP-1 cells and mouse peritoneal macrophages [43]. Vascular aging is related to arterial stiffening, endothelial dysfunction, oxidative stress, and inflammation, leading to atherosclerosis [44]. An aberrant miRNA expression profile is an important element in the process of mammalian aging [45]. In addition, the aberrant expression of age-related miRNAs promotes the onset and development of atherosclerosis [46]. NMN supplementation demonstrates a significant cardiovascular protective effect by enhancing endothelium-dependent vasodilation, mitigating oxidative stress, and ameliorating age-related gene expression in aged mice. Furthermore, the protective effect of NMN on vascular function is associated with anti-aging alterations in the miRNA expression profile in the aged mouse aorta, suggesting that NMN supplementation promotes epigenetic rejuvenation and confers an anti-atherogenic effect [47].

### 3.4. Hypertension

Hypertension is a major risk factor for cardiac, cerebrovascular, and renal diseases [48]. Hypertension is related to aging and obesity, both of which are linked to NAD^+^ deficiency; therefore, NAD^+^ has been considered as a promising therapeutic target for hypertension. NAMPT, a rate-limiting enzyme in NAD^+^ biosynthesis, is significantly downregulated in both patients and experimental animals with hypertension. Moreover, NAMPT knockout (NAMPT^+/−^) mice exhibit significant elevated blood pressure and ROS levels following stimulation with angiotensin II (Ang II) compared to wild-type mice. In contrast, NAMPT overexpression exhibits a protective effect against Ang II-induced hypertension by suppressing the production of ROS [49]. NAM supplementation, a precursor of NAD^+^, significantly prevents blood pressure increases and attenuates the mRNA levels of inflammatory and fibrotic markers in L-NAME-treated mice. Additionally, it also decreases elevated blood pressure in eNOS-null mice, suggesting that NAM has a beneficial effect on hypertension associated with eNOS dysfunction by suppressing inflammation [50]. Furthermore, long-term NAM supplementation contributes to a mild decrease in blood pressure and aortic stiffness in healthy middle aged and older individuals [51]. Although NAM can be converted to NMN by NAMPT, future clinical trials need to investigate the effect of NMN on patients with hypertension.

### 3.5. Arrhythmia

The aberrant metabolism of NAD^+^ in the heart affects the function of the cardiac ion channels. It has been suggested that the NAD^+^/NADH ratio mediates the expression and conductance of cardiac sodium channels (Na(_v_)1.5) by NAD(H)-dependent protein kinase C activation. An elevated NADH level is related to an increased susceptibility to ventricular tachycardia in wild-type mouse heart. Furthermore, NAD^+^ supplementation significantly ameliorates the risk of ventricular tachycardia in mouse heart [52]. NAD^+^ administration significantly restores the cardiac Na(^+^) current in deoxycorticosterone acetate (DOCA)–salt-induced mice with non-ischemic cardiomyopathy, while it improves the conduction velocity in failing human heart, suggesting that NAD^+^ plays a key role in the process of anti-arrhythmia [53]. Cardiac-specific knockout Sirt1 in mice promotes the hyperacetylation of (Na(_v_)1.5) and reduces the (Na(_v_)1.5) level in the cardiomyocyte membrane, leading to cardiac arrhythmia and premature death. Importantly, the arrhythmic phenotype of cardiac-specific knockout Sirt1 in mice recapitulates human cardiac arrhythmia due to the loss of (Na(_v_)1.5) function [54]. The role of NAD^+^ precursors in modulating (Na(_v_)1.5) function have been revealed. NR treatment increases the peak sodium current in a protein kinase C-dependent manner, and it decreases the late sodium current in neonatal rat cardiomyocytes via an acetylation-dependent or -independent mechanism. Additionally, NR supplementation shortens the QT interval in mice to improve cardiac electrophysiology, indicating that NAD^+^ precursors have potential therapeutic effects on cardiac electrophysiology and anti-arrhythmia [55]. NMN, as a crucial NAD^+^ precursor, may mediate the process of anti-arrhythmia, but further clinical trials are needed to explore its effect.

### 3.6. Other Cardiovascular Diseases

Dilated cardiomyopathy (DCM) is characterized by a progressive decline in cardiac contractility and ventricular dilation. Frataxin knockout (FXN-KO) in mice promotes the development of DCM with a reduced ejection fraction [56]. The hearts of FXN-KO mice exhibit mitochondrial protein hyperacetylation, decreased Sirt3 levels, and increased NAD^+^ salvage. Notably, NMN administration improves the cardiac function, extracardiac metabolic function, and energy metabolism in FXN-KO mice in a Sirt3-dependent manner [57].

Cardiac hypertrophy is a complex process in response to various physiologic and pathologic stimuli, and numerous mechanisms are involved in its development, such as cellular metabolism and the immune response [58]. Recently, it has been reported that the NAD^+^ level is significantly reduced in the heart of the transverse aortic constriction (TAC)-induced mice model of cardiac hypertrophy [59]. Moreover, NAD^+^ treatment inhibits the agonist-induced cardiac hypertrophy in vitro and in vivo through the Sirt3-LKB1-AMPK signaling pathway, suggesting that NAD^+^ supplementation is crucial for cardiac hypertrophy treatment [60]. Cardiac-specific conditional knockout Sirt7 in mice exacerbates TAC-induced cardiac hypertrophy and fibrosis, whereas NMN treatment attenuates phenylephrine-induced cardiac hypertrophy, but the change is abolished by Sirt7 knockdown, suggesting that NMN mediates cardiac hypertrophy in a Sirt7-dependent manner [61]. In addition, isoproterenol (ISO) treatment promotes cardiac dysfunction, fibrosis, and hypertrophy in vivo, whereas NMN supplementation alleviates these changes. Furthermore, NMN treatment attenuates the TGF-β-induced activation of cardiac fibroblasts by inhibiting oxidative stress and Smad3 acetylation in a NAD^+^/Sirt1-dependent manner [62].

## 4. NMN Supplementation in Humans

NMN supplementation has demonstrated good efficacy and benefits for cardiovascular diseases both in vivo and in vitro, and several studies have been conducted to investigate its potential clinical applicability. The first human phase I study of NMN supplementation (UMIN000021309) was performed by an international collaborative team between Keio University and Washington University to examine NMN supplementation safety and its effect on human physiological functions [21]. The clinical trial investigated the safety of NMN supplementation in 10 healthy Japanese men. A single-arm, non-randomized trial was conducted to explore the clinical parameters and kinetics of NMN for 5 h after each NMN administration. The Wilcoxon signed-rank test, the mixed-effects model, Bonferroni’s multiple comparison, and Pearson’s correlation analysis were performed for the statistical analyses. The oral administration of NMN (100, 250, and 500 mg) did not result in significant clinical symptoms or alterations in the heart rates, blood pressure, oxygen saturation, body temperature, or sleep quality. Additionally, the laboratory analysis results showed slight alterations in the serum bilirubin, serum creatinine, chloride, and serum glucose levels within a normal range independent of the NMN dosage. Thus, the oral administration of NMN is safe and NMN is effectively metabolized in healthy men without causing acute deleterious effects [63]. The phase II study (UMIN000030609) was conducted to investigate the long-term safety and kinetics of NMN supplementation and its effect on glucose metabolism in healthy subjects [2]. A 10-week, randomized, placebo-controlled, double-blind trial was conducted to investigate the effect of the chronic administration of NMN on the metabolic function in overweight or obese postmenopausal women with prediabetes. A three-way mixed-model analysis of variance (time, condition, and group), Tukey’s post hoc test, and a two-way mixed-model analysis of variance (time and group) were performed for the statistical analyses. The administration of NMN (250 mg) increased the NAD^+^ content in circulating peripheral blood mononuclear cells (PBMCs) and improved muscle insulin signaling, insulin sensitivity, and muscle remodeling in overweight or obese postmenopausal women with prediabetes [64]. Another 24-week, randomized, double-blind study (UMIN000025739) was performed to examine the effect of the long-term administration of NMN (100 mg and 200 mg) on various hormones in healthy subjects [2]. Another 12-week, randomized, double-blind, and placebo-controlled clinical trial (UMIN000036321) was performed to investigate the effect of NMN supplementation (250 mg) on the body composition in elderly subjects [65]. Recently, a new randomized, multicenter, double-blind, placebo-controlled, parallel-group, and dose-dependent clinical trial with the once-daily oral administration of NMN (300, 600, and 900 mg) for 60 days including 80 middle-aged, healthy individuals was conducted. The Wilcoxon signed-rank test, per-protocol analysis, mixed-model analysis, and the paired *t* test were performed for the statistical analyses. NMN supplementation (900 mg) improves the circulating NAD^+^ concentration and is well tolerated and safe. Laboratory analysis and physical examination did not exhibit significant changes for the NMN treatment (300, 600, and 900 mg) over 60 days [66]. The studies on the long-term effects and safety of NMN supplementation suggest that the oral administration of NMN is well tolerated and safe, and it effectively increases NAD^+^ biosynthesis in humans. Long-term NMN supplementation, especially, modestly mitigates postprandial hyperinsulinemia, which is a risk factor of cardiovascular diseases. Thus, the long-term administration of NMN may serve as a novel strategy for the prevention or treatment of cardiovascular disease. These clinical trials on NMN provide a crucial foundation for its potential clinical practice, and intervention with NMN may be a promising strategy for anti-aging, improving glucose metabolism, and even in adjuvant therapy for cardiovascular diseases. However, most clinical trials on NMN nowadays focus on nutraceutical development but not on pharmaceutical development. Although pharmaceutical development for cardiovascular diseases takes quite a lot of time and money, clinical trials on NMN for drug development are necessary in the future.

**Figure 2:**
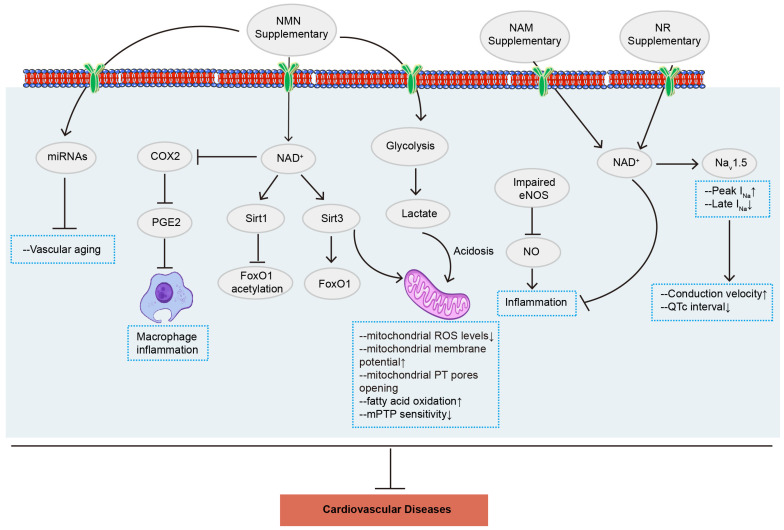
NMN ameliorates cardiovascular diseases via various signaling pathways. NMN and NAD^+^ precursors are potential molecules for the therapy of diverse cardiovascular diseases, including myocardial ischemia/reperfusion injury, heart failure, atherosclerosis, hypertension, arrhythmia, and other cardiovascular diseases. NMN: nicotinamide mononucleotide; NAD^+^: nicotinamide adenine dinucleotide; NAM: nicotinamide; NR: nicotinamide riboside; COX2: cyclooxygenases 2; PGE2: prostaglandin E2; Sirt 1: Sirtuin 1; Sirt 3: Sirtuin 3; FoxO1: Forkhead box O1; eNOS: endothelial nitric oxide synthase; NO: nitric oxide.

Although numerous studies demonstrate the potential mechanism and therapeutic effect of NMN supplementation and NAD^+^ precursors on metabolic and cardiovascular diseases (Figure 2), the clinical and toxicological evidence to support its application remains insufficient. Therefore, further clinical studies are needed to enhance the prospects of NMN as a drug.

## 5. Perspective

The cardiovascular protective effect of NMN has been proposed mainly for its ability to enhance NAD^+^ biosynthesis through a series of enzymatic reactions. The results of the current research indicate that NMN supplementation exhibits potential anti-aging and cardiovascular protective properties. However, only a few clinical studies with small numbers of trial individuals have been conducted, and these studies primarily focus on its safety evaluation rather than its cardiovascular protective effects. NMN, as a nutraceutical, appears to be safe, whereas the pharmacology of NMN in vivo is complex, and our understanding about it remains limited. Thus, systematical evaluation of NMN supplementation still needs to be conducted. Additionally, because the treatment responses and cardiovascular disease symptoms differ between males and females, investigating potential gender-specific variations in the effectiveness of NMN in further animal experiments and clinical trials will help to better understand its impact on cardiovascular disease treatment. In addition to the therapeutic effect of NMN administration alone, future research should also investigate its potential effect on cardiovascular disease treatment as an adjuvant to other drugs.

Notably, NMN is currently served as a nutraceutical, but we should still be cautious until sufficient evidence regarding its safety and efficacy is available. Moreover, NMN supplementation may be not suitable for all populations. For example, NMN supplementation protects against myocardial I/R injury by enhancing glycolysis and acidosis, but the acidic condition stimulates tumor angiogenesis and expedites tumor cell growth [35,67]. Several antineoplastic drugs inhibit DNA repair and angiogenesis to induce tumor cell apoptosis by targeting enzymes involved in the salvage pathway [68,69,70]. However, NMN supplementation restores the NAD^+^ level and tumor cell viability and reduces antineoplastic drug-induced tumor cell apoptosis [69,70]. Nevertheless, it is also reported that exogenous pyruvate promoting NAD^+^ biosynthesis inhibits tumor growth [71]. Thus, further studies are needed to investigate the effect of exogenous NMN supplementation on tumor patients.

## 6. Conclusions

Taken together, NMN supplementation acts as a promising approach for improving cardiovascular metabolism health and therapeutic cardiovascular diseases. However, further clinical trials are still needed to explore the mechanism of cardiovascular protection, appropriate population, and optimal dosage for NMN supplementation.
